# Selenium-containing nanoparticles and bone related disorders: from synthesis, bone metabolism to disease therapy

**DOI:** 10.3389/fendo.2025.1724888

**Published:** 2025-12-12

**Authors:** Ziyang Yue, Haonan Li, Yi Hu, Jinshen Shen

**Affiliations:** 1 Hubei Key Laboratory of Tumor Microenvironment and Immunotherapy, China Three Gorges University, Yichang, China; 2 College of Basic Medical Sciences, China Three Gorges University, Yichang, China; 3 Yichang Key Laboratory of Infection and Inflammation, China Three Gorges University, Yichang, China

**Keywords:** bone-related diseases, nanomedicine, osteoclastogenesis, osteogenesis, selenium nanoparticles

## Abstract

Bone-related disorders (BRDs), such as osteoporosis, rheumatoid arthritis, and osteosarcoma, are major contributors to morbidity and disability worldwide. Conventional treatments are often limited by poor targeting, systemic toxicity, and insufficient long-term efficacy. Selenium (Se), an essential trace element, plays a pivotal role in maintaining redox homeostasis, immune balance, and bone remodeling. Selenium-containing nanoparticles (SeNPs) have emerged as promising platforms that integrate the biological activities of selenium with the tunable features of nanomaterials. This review provides a comprehensive overview of SeNPs, covering synthesis strategies, physicochemical properties, and their roles in regulating osteogenesis, osteoclastogenesis, oxidative stress, and inflammatory signaling in the skeletal microenvironment. We further highlight recent advances in applying SeNPs for the treatment of BRDs, including their incorporation into biomaterials and combination therapies such as photothermal and chemodynamic approaches. While preclinical studies show encouraging results, challenges remain in understanding long-term biosafety, biodistribution, and clinical translation. Overall, SeNPs-based nanomedicine offers significant potential for precision bone-targeted therapies and tissue regeneration.

## Highlights

- SeNPs demonstrate dual regulatory capacity by promoting osteoblastic bone formation through BMP-2/Smad/Runx2 signaling while suppressing osteoclastogenesis via modulation of ROS/NF-κB/NFATc1 pathways.
- Multifunctional SeNPs-based platforms enable synergistic therapy through integration with biomaterials and multimodal strategies such as photothermal and chemodynamic therapy, offering targeted intervention for bone-related disorders with enhanced bioavailability and reduced systemic toxicity.

## 1 Introduction

Bone related disorders (BRDs), encompassing such as rheumatoid arthritis (RA), osteoarthritis (OA), osteosarcoma (OS) and bone tumors pose a sustained threat to musculoskeletal integrity across the lifespan and are closely linked to elevated morbidity and mortality. Existing therapeutic modalities for bone diseases often exhibit suboptimal outcomes and off-target toxicities (1, 2). Furthermore, they rarely address the intricate crosstalk among pathologic bone resorption, diminished osteogenesis, and persistent inflammatory signaling. These shortcomings necessitate the development of advanced nanotherapeutics that integrate biomaterial engineering, redox regulation, and cell-specific targeting to re-establish bone homeostasis (3).

Selenium (Se), a vital micronutrient, is indispensable for maintaining physiological homeostasis and has been increasingly implicated in the pathogenesis of osteoporosis (OP) (4). Upon dietary absorption, selenium is predominantly processed in hepatic tissues and exerts its physiological influence via selenoproteins—a unique family of redox-active biomolecules with potent anti-inflammatory and antioxidant properties (5, 6). To date, 25 human genes encoding selenoproteins have been identified, reflecting their evolutionary conservation in cellular regulatory networks (7). Both deficiency and excess of Se are associated with adverse outcomes: while inadequate levels correlate with increased mortality, immune dysregulation, and neurocognitive decline, supraphysiological concentrations may induce toxicity; thus, a narrow therapeutic index governs its clinical utility (8).

Critically, the therapeutic application of selenium is constrained by a narrow concentration window, as its role shifts from an essential micronutrient to a potential toxin at slightly elevated doses. In contrast, selenium-containing nanoparticles (SeNPs) demonstrate markedly attenuated systemic toxicity, superior bioavailability, and extended circulatory half-life compared to traditional Se formulations (9, 10). These physicochemical and pharmacokinetic enhancements render SeNPs a promising vector for targeted theragnostic. Mechanistic investigations have revealed their capacity to modulate diverse pathological processes, including oncogenesis, microbial infections, viral replication, hepatic dysfunction, metabolic disorders, and neurodegeneration (11, 12). Emerging research focused on decoding selenium’s osteoprotectory role and advancing SeNPs-based interventions toward clinical application. A key challenge involves systematic characterization of structure– function relationships across SeNPs variants and rigorous validation of therapeutic efficacy in disease-relevant preclinical models (7). This paradigm underscores the imperative to optimize selenium-based interventions within the constraints of physiological homeostasis.

This review synthesizes cutting-edge advances in SeNPs-mediated therapies for BRDs, emphasizing their multimodal roles in redox regulation, inflammatory suppression, and bone anabolic signaling. This review deliberately emphasizes research progress from 2018 to 2025, supplemented by a limited number of earlier seminal studies to frame the development of the field. Accumulating studies underscore SeNPs’ capacity to modulate redox balance (13), suppress osteoclastogenesis (14), and promote osteoblast activity (15, 16), thereby exhibiting therapeutic potential in OP, OS, RA and OA. Importantly, the synergistic integration of SeNPs with biomaterials (e.g., hydrogels, scaffolds) and therapies (e.g., photothermal/chemodynamic therapy) represent a novel strategy in bone-targeted nanomedicine (17). Nevertheless, several challenges remain, including elucidation of long-term biodistribution, the optimization of dose-dependent responses, and development of standardized clinical-grade synthesis protocols. Future investigations should focus on mechanistic insights, protocols standardization, and translational validation to unlock the full clinical potential of SeNPs in precision bone therapeutics.

## 2 Synthesis of SeNPs

Previous reviews, such as the one by Bisht et al. (18), have summarized general synthesis strategies for SeNPs. Building on this foundation, the present review focuses specifically on Se-containing nanoparticles in the context of bone-related disorders (18). In recent years, SeNPs have garnered escalating attention owing to their unique properties and expanding repertoire of biomedical utilities. To harness these benefits, a spectrum of synthetic strategies —spanning physical, chemical, and biological paradigms—has been meticulously optimized to yield SeNPs with enhanced colloidal stability and functional versatility (19).

Among these, chemical synthesis remains the most extensively adopted route, primarily involving the reductive transformation of selenium precursors (e.g., sodium selenite) using ascorbate, monosaccharides, or hydrazine as electron donors (20). The choice of reducing and stabilizing agents significantly influences the physicochemical properties of the resulting SeNPs, including their size, shape, and biological activity (21). Niu et al. introduced a hydrothermal approach that rapidly gained widespread adoption due to its operational simplicity, scalability, and cost-effectiveness (22). Boroumand et al leveraged ascorbic acid reduction in the presence of polyvinyl alcohol (PVA) or chitosan to synthesize spherical SeNPs (≈50–70 nm), demonstrating potent antioxidant activity (DPPH clearance >90%) and antibacterial properties (23). A subsequent study revealed that SeNPs synthesized via chemical reduction by cysteine or ascorbic acid, with PVA stabilization, yielded particles whose size, morphology, and antioxidant capacity were strongly influenced by purification protocols (24). More recently, Siddique et al demonstrated an ascorbic acid–mediated green chemical route to produce SeNPs with notable antihyperuricemic, antioxidant, anticoagulant, and thrombolytic activity, underscoring their multifaceted biomedical potential (25).

Conversely, physical methods such as deposition and laser ablation offer reagent-free synthesis routes that yield high-purity SeNPs with precise size dispersity. For instance, Guisbiers et al. synthesized pure SeNPs (~115 nm) by pulsed laser ablation in deionized water, which showed dose-dependent inhibition (~50 ppm) against E. coli and *S. aureus* with high purity and low cytotoxicity (26). Menazea et al. successfully synthesized doped SeNPs via single-step laser ablation, enhancing their antibacterial activity (27). More recently, laser ablation in organic solvent (isopropanol) yielded crystalline Se nanorods (~200 nm thick, 2–4 μm long), offering tunable morphology beyond typical spherical SeNPs (28). Nevertheless, the energy-intensive nature and limited batch throughput physical methods constrain their widespread adoption.

Eco-compatible biosynthesis, capitalizing on leveraging plant and microbial reduction, yields superior SeNPs with non-toxicity, cost-efficiency, stability, and biocompatibility, offering promising potential for anti-bacterial and anti-cancer treatments (20). *Bacillus paramycoides* can catalyzes selenite reduction under mild conditions (37°C, pH = 6) achieving ~99% conversion efficiency; the resulting SeNPs inhibited *Staphylococcus aureus* and *E. coli* at minimum inhibitory concentrations (MICs) of ~400–600 µg/mL, along with strong antioxidant activity (29). Similarly, SeNPs biosynthesized from *Streptomyces parvulus* MAR4 and SeNP−chitosan nanocomposites exhibited significant antimicrobial and anticancer potential (30). In another approach, Drimia indica leaf extract was employed to fabricate DI−SeNPs that induced apoptosis in A549 lung cancer cells and demonstrated broad-spectrum antimicrobial efficacy (31). Continued refinement of these green synthetic protocols will be pivotal in establishing SeNPs as a versatile nanotherapeutic platform for bone-related disorders BRDs (**Table 1**).

**Table 1:** Comparison of representative SeNPs synthesic methods.

Method	Core steps	Advantages	Disadvantages	Application scenarios	References
Chemical	Reduction with agents (e.g., vitamin C)	Cost-effective, scalable	Broad size distribution	Lab-scale synthesis	(20)
Hydrothermal (physical)	High-temperature/pressure reaction	Uniform morphology, good yield	Complex equipment	Industrial production	(22)
Template-assisted (physical)	Stabilizer-guided growth	Controlled shape/size	Template removal required	Specialized morphology	(21)
Laser ablation (physical)	Laser irradiation	Monodisperse, high purity	Equipment-intensive	Not widely used	(27)
Biosynthesis	Plant/microbial reduction	Eco-friendly, biocompatible	Low purity	Biomedical coatings	(20)

## 3 Roles of SeNPs in bone metabolism

Selenium-containing nanoparticles (SeNPs) exert multifaceted effects on bone metabolism, and their antioxidant/redox activity is a central mechanism that operates across different cell types and regulatory pathways, enabling selective modulation of osteoclasts, osteoblasts, and the bone-immune niche. SeNPs enhance osteogenesis and suppress osteoclast-mediated bone resorption primarily through modulation of key bone signaling pathways and immunoregulation within the bone microenvironment, positioning them as promising nanoplatforms for restoring bone homeostasis under pathological conditions.

### 3.1 Inhibition of osteoclastogenesis

Excessive osteoclast activity drives progressive bone loss in various skeletal disorders. Among SeNPs, Lentinan-selenium nanoparticles (LNT-Se) have shown potent inhibition of osteoclastogenesis by reducing ROS levels and downregulating NF-κB and NFATc1, thereby suppressing osteoclast-specific gene expression and bone resorption (32). Another research indicated that SeNPs mitigate anastrozole-induced bone toxicity by reducing osteoclastogenesis and enhancing osteoblast activity, as validated in both HOS cell cultures and SD rat models, thereby preventing bone density loss and estrogen deficiency-related OP (33). Furthermore, Sharma et al. synthesized a SeNPs-based Qu nanoformulation (Qu-SeNPs), which demonstrated effective reduction of the RANKL/OPG ratio in osteoblasts and significant suppression of osteoclastogenesis (16).

Taken together, these convergent data underscore the capacity of SeNPs to restrain osteoclast number and function, attenuate net bone resorption, and thereby sustain skeletal mass under pathological challenge.

### 3.2 Promotion of osteogenesis

Osteoblast-mediated bone formation is tightly regulated by redox balance and inflammatory cues (34), rendering these cells particularly susceptible to therapeutic modulation by SeNPs.

SeNPs have been reported to promote osteoblast differentiation by regulating the activity of alkaline phosphatase (ALP) and promoting the formation of calcium nodules as well as increasing collagen protein content (15). Selenium treatment improves osteoblast differentiation of bone marrow mesenchymal stem cells (BMSC) and protects MSCs from H_2_O_2_ inhibition of osteoblast differentiation by inhibiting oxidative stress and extracellular signal-regulated kinase (ERK) activation (23). In addition, SeNPs exhibit potent antioxidant properties by scavenging excess intracellular ROS, thereby protecting osteoblasts from oxidative damage and preserving their functional capacity under stress conditions (35). Similarly, Lee et al. demonstrated that SeNPs safeguard LPS-treated MC3T3-E1 pre-osteoblasts from apoptosis and functional impairment by enhancing cellular adhesion and maintaining osteogenic potential, primarily through activation of the PI3K/Akt signaling pathway (36).

Collectively, these multi-pronged mechanisms—spanning redox re-calibration, anti-inflammatory signaling, and pathogen-restrictive actions—establish SeNPs as an integrative platform for next-generation bone regenerative therapies applications (37).

### 3.3 Regulation of bone metabolism by SeNPs

In addition to the well-established regulation of BMP-2/Smad and Runx2 transcriptional networks (15, 38), SeNPs exert their effects on bone metabolism via a spectrum of additional mechanisms. As potent antioxidants, SeNPs scavenge excessive ROS, mitigating oxidative stress-induced damage to osteoblasts and BMSCs, thereby preserving their proliferation and differentiation capacity (35). Maintaining such redox equilibrium is indispensable, as unchecked oxidative pressure remains a principal instigator of catabolic bone turnover.

**Figure 1:**
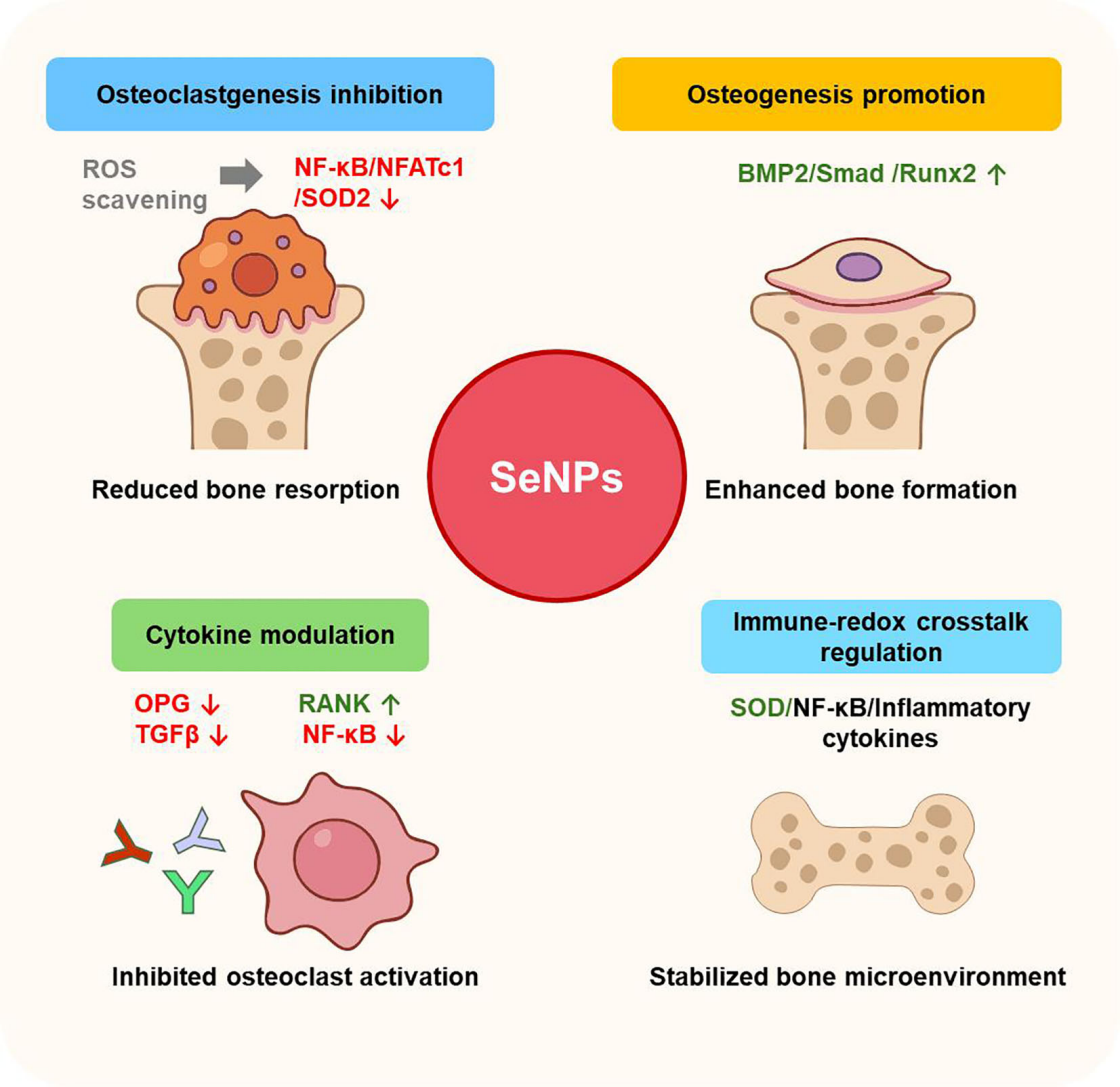
Multifunctional roles of SeNPs in bone-related disorders. SeNPs inhibit osteoclastogenesis via ROS scavenging and downregulation of NF-nr and NFATc1, while promoting osteoblastogenesis through activation of BMP2/Smad/Runx2/Nrf2 pathways. They also modulate the OPG/RANKL axis to suppress osteoclast activation. Additionally, SeNPs restore redox balance and regulate immune signaling, thereby stabilizing the bone microenvironment.

Beyond radical scavenging, SeNPs finely tune the cytokine milieu within the osseous niche. They upregulate osteoprotegerin (OPG) secretion while downregulating receptor activator of nuclear factor κB ligand (RANKL) expression, effectively shifting the OPG/RANKL ratio to inhibit osteoclastogenesis and bone resorption (16). SeNPs synthesized by Lactobacillus casei exhibit potent anti-inflammatory and antioxidative effects through modulation of key markers, including NF-κB, TGF-β, Nrf2, and SOD2 in inflamed Caco-2 cells, underscoring their therapeutic potential (39, 40). Notably, Xia et al. demonstrated through integrated serum proteomics and transcriptomic analyses in zebrafish that SeNPs exert broad immunomodulatory effects beyond redox regulation, acting on both healthy and diseased states (41). They identified SOD and NF-κB as pivotal molecular switches linking SeNPs-mediated redox activity with immune responses, suggesting that immune-redox crosstalk may underline SeNPs-driven regulation of bone homeostasis (**Table 2**). These convergent mechanisms—oxidative stress containment, cytokine re-balancing, and immune recalibration—are schematically integrated in **Figure 1**.

**Table 2:** Mechanistic classification of SeNPs in bone remodeling.

Action direction	Key targets	Molecular mechanism	Biological effect	References
Osteoclastogenesis inhibition	NF-κB/NFATc1/SOD2	ROS scavenging → Inhibition of transcription factors (NF-κB/NFATc1)	Reduced bone resorption	(16, 32)
Osteoblastogenesis promotion	BMP2/Runx2/Smad	Activation of Smad signaling → Upregulation of ALP/collagen	Enhanced bone formation	(15, 23, 34, 37)
Cytokine modulation	OPG/RANKL/TGF-β/NF-κB	Upregulation of OPG, downregulation of RANKL → Blockade of RANKL-RANK signaling	Inhibited osteoclast activation	(16, 39, 40)
Immune-redox crosstalk regulation	SOD/NF-κB/inflammatory cytokines	SeNPs-mediated redox balance ↔ modulation of immune signaling pathways	Stabilized bone microenvironment; dual antioxidant and anti-inflammatory effects	(39–41)

### 3.4 Comparison with other biomaterials in BRDs

In addition to selenium−based nanoparticles (SeNPs), a variety of other nano−elements and nanomaterials have been explored for bone regeneration, bone repair or treatment of bone−related disorders. Traditional nano-elements for bone repair, such as zinc-doped hydroxyapatite(Zn-HA) (42), zinc/strontium-codoped hydroxyapatite (ZnSr-HA) (43) and bioactive glass (BG) scaffolds (44), mainly function via osteoconductivity, ion release, and structural support, promoting osteoblast proliferation, differentiation, and mineralization. Although these materials are effective for bone regeneration and mechanical reinforcement, they do not directly modulate oxidative stress, inflammation, or immune activity.

For instance, Zn−containing bioactive glass membranes have been shown to enhance viability and alkaline phosphatase (ALP) activity in osteoblastic Saos-2 cells, supporting osteoconduction and bone cell proliferation (45–47). Additionally, Zn-based biodegradable metals and implants have been reviewed as promising materials for fracture healing, critical-size bone defect repair, and alveolar bone regeneration, with the release of Zn^2+^ ions promoting bone repair and regeneration through mechanisms including osteogenesis, angiogenesis, and inhibition of excessive bone resorption (48). Zn−containing bioactive glass–hydrogel composite demonstrated accelerated bone defect healing in rats, accompanied by enhanced angiogenesis (new blood vessel formation) and osteogenesis, indicating that Zn-based materials can support both bone formation and vascularization — a key aspect for bone repair in large or complex defects (49). Reviews on Zn-based biomaterials also emphasize that Zn incorporation can promote osteoblast proliferation, differentiation, and mineral deposition, while simultaneously inhibiting osteoclast activity to maintain bone homeostasis (50, 51).

However, compared to these Zn-based (or other inorganic) nano-materials, SeNPs offer additional functionalities beyond structural support or ion release. SeNPs have been experimentally demonstrated to promote MSC/osteoblast differentiation and osteogenesis under oxidative stress while simultaneously inhibiting osteoclastogenesis (16, 32, 35, 41). These findings indicate a clear functional distinction: SeNPs offer biochemical, metabolic, immunomodulatory, and redox-related benefits, whereas Zn/HA-based materials provide advantages in structural support, bone guidance, ion delivery, and mechanical stability.

## 4 SeNPs in bone-related disorders

### 4.1 Osteoporosis

Osteoporosis (OP) is a prevalent metabolic bone disorder marked by decreased bone mass and microarchitectural deterioration, leading to increased fracture risk (70). Epidemiological data indicate that approximately 10.2% of adults over 50 are affected, with projections rising to 13.6% by 2030 (71). Given the limited efficacy and poor patient adherence associated with current bisphosphonate-based therapies, there is an urgent demand for innovative interventions. These should target osteoblast activation, osteoclast suppression, and inflammatory modulation—particularly via advanced platforms such as nanoparticle delivery systems.

Fatima et al. demonstrated that low concentrations of SeNPs (50 ng/mL) promote human mesenchymal stem cell (hMSC) differentiation into osteoblasts by mitigating oxidative stress. This enhancement includes improved cell viability, ALP activity, enhanced mineralized nodule formation, and suppressed adipogenic gene expression. The JNK/FOXO3a signaling pathway was shown to mediate these effects by upregulating antioxidant enzymes (e.g., SOD, catalase). However, higher SeNP concentrations (300 ng/mL) induce cytotoxicity due to excessive ROS production (35). What’s more, SeNPs activate the BMP-2/MAPK/β-catenin pathway, which is crucial for osteoblast differentiation, while simultaneously attenuating inflammatory responses via suppression of NF-κB/NFATc1. These dual actions contribute to the regulation of osteoblast–osteoclast balance, particularly in pathological settings like diabetes-associated OP. In a type 2 diabetes-induced OP rat model, SeNPs preserved trabecular architecture, improved bone strength (e.g., maximum load and stiffness), and restored bone mineral density (15). Another study reported that SeNPs suppress osteoclastogenesis by upregulating selenoproteins like GPx1, reducing ROS levels, and reprogramming macrophage polarization from M1 (pro-inflammatory) to M2 (pro-regenerative) states. This redox-sensitive immune modulation underpins SeNPs’ ability to reduce bone resorption while supporting regeneration, forming a dual-action therapeutic model for OP (52).

Elmala et al. showed that SeNPs improved collagen synthesis and osteoblast function in an anastrozole-induced OP rat model. Compared with Nano Vitamin D3, SeNPs led to greater collagen area and higher osteoblast counts in alveolar bone (53). Similarly, Vekariya et al. found that SeNPs attenuated anastrozole-induced osteoclast activation and preserved bone density in Sprague-Dawley rats (33). In ovariectomized rats, SeNPs also prevented estrogen deficiency-induced bone loss, reinforcing their therapeutic applicability (53).

Beyond therapeutics, Byun et al. introduced selenium-modified CdZnTe (CZTS) detectors for bone mineral density (BMD) quantification. CZTS demonstrated high stability and accuracy in BMD estimation (1.1972 g/cm² vs. actual 1.2 g/cm²), suggesting feasibility for clinical OP diagnostics (72). Although not directly linked to OP treatment, Ruan et al. demonstrated that SeNPs incorporated into chitosan-ZnO scaffolds enhanced re-epithelialization and collagen synthesis during pediatric fracture wound healing (73).

Collectively, SeNPs provide a multi-pronged platform—augmenting osteogenesis, restraining osteoclast activity, reprogramming immune responses, and preserving micro-architectural integrity—offering a blueprint for personalized, minimally invasive management of OP and allied BRDs.

### 4.2 Rheumatoid arthritis

Rheumatoid arthritis (RA) is a chronic systemic autoimmune disorder characterized by persistent symmetrical joint inflammation, leading to cartilage degradation, bone erosion, and eventual disability (74). Extra-articular manifestations, such as atherosclerosis, myocardial infarction, and heart failure, further exacerbate the disease burden. According to the Global Burden of Disease Study, RA affects approximately 1% of the global adult population and is three to four times more prevalent in women than in men (54, 75). Despite the advent of biologic agents and targeted synthetic therapies, challenges including partial remission, drug resistance, and systemic toxicity persist, necessitating the exploration of innovative therapeutic strategies.

SeNPs demonstrate potent antioxidant effects by enhancing endogenous antioxidant enzymes such as glutathione peroxidase (GPx), superoxide dismutase (SOD), and thioredoxin reductase (TrxR). These enzymes effectively scavenge reactive oxygen and nitrogen species (ROS/RNS), thereby reducing oxidative damage in inflamed synovial tissues. Concurrently, SeNPs neutralize free radicals (e.g., hydrogen peroxide, superoxide anions), further alleviating oxidative stress (76–78). Simultaneously, SeNPs inhibit pro-inflammatory cytokines (TNF-α, IL-6, IL-1β) via suppression of NF-κB signaling. *In vivo* studies confirm reduced paw swelling and joint damage through restored antioxidant levels and anti-inflammatory effects (56, 79, 80).

Beyond general anti-inflammatory effects, SeNPs also target pathological neovascularization and synovial inflammation through NO-mediated endothelial apoptosis and AMPKα/mTOR-autophagy pathways, leading to reduced NF-κB activity and cartilage (7, 57, 81).

Recent advances in nanotechnology have led to the development of multifunctional SeNP-based delivery platforms with enhanced therapeutic efficacy against RA. One such example is Pd@Se-HA nanoparticles, which integrate photothermal therapy (PTT) with intrinsic antioxidant capacity. These nanoparticles significantly inhibit pro-inflammatory cytokines and prevent joint destruction, while maintaining low systemic toxicity (55).Another innovative approach involves Pd@MSe-TPP nanomotors, which exhibit autonomous movement and specifically target mitochondria within macrophages. By restoring redox balance in inflammatory cells, these nanomotors effectively suppress oxidative stress and safeguard cartilage from degenerative changes (82). Moreover, CdSe mesoporous quantum dots (MSQDs) have demonstrated strong antioxidant activity in both neutrophils and macrophages. Their use has been associated with a marked reduction in inflammatory responses and attenuation of RA progression (83). In addition to synthetic designs, green synthesis strategies have also been explored. Fennel-derived SeNPs, produced through eco-friendly biosynthetic routes, possess excellent biocompatibility and potent anti-inflammatory activity. These naturally sourced nanoparticles have shown promise in slowing RA progression and preserving cartilage integrity (84).

Collectively, SeNPs offer multifaceted benefits for RA management. They not only alleviate oxidative stress and inhibit inflammatory cytokines but also modulate immune responses. SeNPs may also reduce autoantibody titers, including rheumatoid factor (RF), thereby suppressing synovial hyperplasia and joint degradation. Their integration into bioengineered nanoplatforms opens avenues for targeted, low-toxicity, and patient-specific therapies in RA.

### 4.3 Osteoarthritis

Osteoarthritis (OA) is a chronic joint disorder characterized by progressive degeneration of articular cartilage, osteophyte formation at joint margins, and synovial inflammation (58, 85). The disease progression reflects an imbalance between cartilage degradation and repair, resulting in joint pain, stiffness, functional impairment, and ultimately disability (86). Current therapeutic options, such as nonsteroidal anti-inflammatory drugs (NSAIDs) and intra-articular hyaluronic acid (HA) injections, primarily offer symptomatic relief but fail to halt or reverse cartilage deterioration. Therefore, there is an urgent need for innovative disease-modifying treatments.

Recent studies have highlighted the potential of SeNPs in OA management. Ruan et al. summarized that SeNPs effectively mitigate inflammation and oxidative damage in OA by scavenging ROS and enhancing selenoprotein synthesis, demonstrating promising therapeutic efficacy with favorable safety profiles (87). Hu et al. further developed SeNP-incorporated injectable hydrogels that restore redox balance through GPX1 activation, ROS clearance, suppression of matrix metalloproteinase-13 (MMP13), and inhibition of chondrocyte apoptosis in OA rat models. This nanozyme-based approach attenuates cartilage degradation, subchondral bone sclerosis, and synovitis, suggesting a novel antioxidant strategy for OA treatment (59).

Moreover, SeNPs have been shown to suppress inflammation, oxidative stress, and extracellular matrix (ECM) degradation via modulation of the NF-κB/p38 signaling pathways in both *in vitro* and *in vivo* studies (88). Liu et al. demonstrated that targeted delivery of SeNPs using hydrogel microspheres effectively scavenges ROS, alleviates oxidative stress, and preserves cartilage integrity, leading to improved mitochondrial function and joint health (89). Additionally, Han et al. reported that selenium-chondroitin sulfate (SeCS) nanoparticles synthesized via ultrasonic/dialysis methods exhibited lower cytotoxicity compared to sodium selenite. *In vitro*, SeCS significantly reduced T-2 toxin-induced chondrocyte apoptosis relative to chondroitin sulfate alone, indicating potential for treating OA and Kashin-Beck disease (KBD) (90).

Another promising approach involved SeNPs embedded in polycaprolactone (PCL) scaffolds, which reduced ROS levels and inflammation while promoting chondrocyte proliferation in OA rabbit models. This composite scaffold facilitated meniscal repair and improved joint function, demonstrating its potential as a regenerative therapy for OA (60).

In summary, SeNPs confer robust antioxidant and anti-inflammatory actions that attenuate oxidative stress and dampen inflammatory signaling within osteoarthritic cartilage. Through suppression of key pro-inflammatory cytokines—specifically interleukin-1β (IL-1β) and tumor necrosis factor-α (TNF-α)—SeNPs inhibit chondrocyte apoptosis and stimulate extracellular matrix synthesis. These integrated effects translate into reduced joint pain, improved functional performance, and slower progression of OA.

### 4.4 Osteosarcoma

Osteosarcoma (OS) manifests as a highly invasive skeletal neoplasm with peak incidence in adolescence and early adulthood. It poses significant clinical challenges due to its high metastatic potential—especially to the lungs—and generally poor survival outcomes (91, 92). Current therapeutic regimens typically involve a multimodal approach combining surgical tumor resection, chemotherapy, and radiotherapy (93, 94). Despite some efficacy, these conventional treatments are often limited by systemic toxicity, adverse side effects, and high recurrence rates (62). SeNPs, as emerging nanomedicine agents, have demonstrated encouraging therapeutic outcomes in improving OS treatment outcomes.

Several studies report that SeNPs can enhance the activity of antioxidant enzymes such as superoxide dismutase (SOD), catalyzing the conversion of superoxide anion radicals (·O_2_^−^) into hydrogen peroxide (H_2_O_2_). This enzymatic activity elevates H_2_O_2_ levels within the tumor microenvironment, thereby potentiating chemodynamic therapy (CDT) through increased ROS generation, which selectively induces tumor cell apoptosis (61, 95, 96). Additionally, the intrinsic antibacterial properties of SeNPs against both Gram-positive and Gram-negative bacteria may confer benefits in preventing infections during OS therapy (97).

Beyond monotherapies, combination strategies involving SeNPs have been extensively explored. Khan et al. demonstrated that carbon-coated selenium-hydroxyapatite (CC/Se-HAp) nanocomposites exhibit superior anticancer efficacy compared to Se-HAp nanoparticles alone (65, 67). These nanocomposites, with sub-100 nm size, show enhanced cellular internalization via improved membrane penetration, while maintaining a favorable cytotoxicity profile (67). In OS models, CC/Se-HAp selectively targets tumor cells through ligand-mediated interactions and spares healthy mesenchymal progenitors (66).

Moreover, Lei et al. reported that doxorubicin (DOX) loaded on selenium-doped mesoporous silica nanoparticles (SeMS) enhances treatment specificity and reduces side effects compared to free DOX. The SeMS are surface-modified with HA, a natural polysaccharide that serves as a pH-sensitive gatekeeper and targets the CD44 receptor overexpressed on OS cells, enabling controlled and tumor-selective drug release (12, 63, 64, 98). Consequently, DOX-loaded SeMS represent a promising platform for systemic and localized OS therapy.

Numerous additional studies have highlighted multifunctional selenium-based nanoplatforms such as selenium/magnesium ferrite layered double hydroxide nanosheets, which combine antitumor, antibacterial, and bone-regenerative capabilities (99–101). Selenium-doped mesoporous silica nanoparticles have also been shown to mitigate toxicity toward osteoblasts, addressing a key safety concern (102). Furthermore, combining highly active selenium nanotherapeutics with metformin has demonstrated synergistic enhancement of natural killer (NK) cell-mediated antitumor immunity in OS models (103–105).

In summary, SeNPs exerts multi-pronged anti-OS activity—direct cytotoxicity, chemosensitization, redox rebalancing, inflammation restraint, angiogenesis blockade, and immune reprogramming—yet rigorous long-term safety, biocompatibility, and pharmacokinetic profiling remain imperative for clinical translation.

### 4.5 Breast cancer bone metastasis

Breast cancer can often spread to bone, creating osteolytic lesions that result in bone pain, fractures, and poor quality of life. The vicious cycle of bone destruction is driven by increased osteoclast activity and impaired osteoblast function within the bone microenvironment, facilitating tumor growth and skeletal complications (106). Conventional treatments such as bisphosphonates and RANKL inhibitors aim to reduce bone resorption but face challenges including drug resistance and systemic side effects (107).

Recent studies have demonstrated that SeNPs not only exhibit anti-tumor effects by inducing apoptosis and inhibiting proliferation of cancer cells but also modulate the bone microenvironment to inhibit osteoclastogenesis and bone resorption (69). For instance, Li et al. showed that SeNPs effectively suppress osteoclast differentiation and bone degradation in breast cancer bone metastasis models, thereby limiting tumor-induced bone destruction (14).

Moreover, multifunctional nanoplatforms combining Se-based materials with other therapies have been developed to enhance treatment efficacy. Zou et al. reported an indocyanine green (ICG)-loaded Cu_2_-xSe-based ZIF-8 nanoplatform that integrates photothermal therapy and CDT, achieving synergistic anti-tumor effects in a murine breast cancer bone metastasis model, significantly reducing tumor burden while preserving bone integrity (68) (**Table 3**).

**Table 3:** Therapeutic applications of SeNPs in bone disorders.

Disease type	SeNP formulation	Synergistic strategy	Therapeutic outcome	Animal model	References
Osteoporosis	LNT-Se nanoparticles	Activation of JNK/FOXO3a pathway	Improved bone density, reduced adipogenesis	OVX rats	(23, 24)
	SeNPs (low dose)	Antioxidant modulation via BMP-2/MAPK/β-catenin pathway	Promoted osteoblastogenesis, reduced osteoclastogenesis	Diabetic rat model	(15, 52)
	SeNPs vs Nano-Vitamin D3	Collagen synthesis & osteoblast activation	Enhanced bone collagen, osteoblast count, reduced bone loss	Anastrozole rat model	(33, 53)
Rheumatoid arthritis	ICG@Cu_2_-XSe-ZIF-8	Photothermal/chemodynamic synergy	Suppressed joint erosion, inhibited IL-6	CIA mice	(20, 54)
	Pd@Se-HA NPs	Antioxidant + photothermal therapy	Inhibited joint destruction, reduced cytokines	RA rats	(55)
	SeNPs (various formulations)	NF-κB inhibition, NO production, AMPKα/mTOR regulation	Reduced inflammation, angiogenesis, and oxidative stress	RA rats, CIA mice	(7, 56, 57)
Osteoarthritis	Se-CS hydrogel	Antioxidant + MMP13 inhibition	Cartilage repair, reduced synovitis	Rabbit OA model	(58, 59)
	SeNPs in PCL scaffolds	ROS scavenging, chondrocyte proliferation	Reduced inflammation, promoted meniscal repair	OA rabbit model	(60)
Osteosarcoma	Se@HA-CD44 NP	Targeted delivery of Dox	Induced apoptosis, inhibited metastasis	OS cell lines	(61, 62)
	SeMS (HA-modified Se-mesoporous silica)	pH-responsive drug delivery (DOX)	Targeted cytotoxicity, reduced systemic side effects	OS mouse model	(63, 64)
	CC/Se-HAp nanocomposite	Membrane penetration & ROS induction	Enhanced tumor killing, sparing healthy cells	OS cell lines	(65–67)
Bone metastasis (BrCa)	Cu_2_-xSe@ZIF-8/ICG nanoplatform	Photothermal + CDT	Reduced tumor burden, preserved bone integrity	Breast cancer model	(68)
	SeNPs	Anti-osteoclastogenesis, ROS modulation	Suppressed bone resorption, inhibited tumor-induced bone loss	Mouse bone metastasis	(14, 69)

SeNPs attack tumor cells while rebalancing bone turnover, positioning it as a potential therapy for breast-cancer bone metastasis; preclinical optimization of delivery, specificity, and safety is required.

## 5 Conclusion and prospects

SeNPs have emerged as a multifunctional nanoplatform with significant potential in bone regenerative medicine and disease management (17, 108). Their unique ability to modulate redox balance, inhibit osteoclastogenesis, and promote osteogenesis positions them as a promising therapeutic strategy for OP, OA, RA, and OS. By activation of selenoprotein, SeNPs effectively scavenge reactive oxygen species (109), suppress pro-inflammatory pathways (110, 111), and reprogram immune responses, thereby addressing both pathological bone resorption and impaired osteogenesis. Furthermore, their synergistic integration with biomaterials and combination therapies—such as photothermal/chemodynamic approaches—has demonstrated enhanced efficacy in preclinical models, offering a foundation for precision nanomedicine in musculoskeletal disorders (12).

SeNPs still face key obstacles before reaching the clinic. Critical gaps include elucidating long-term biodistribution and toxicity profiles, standardizing synthetic protocols to ensure reproducibility, and validating clinical-grade formulations (2). Future work should pinpoint mechanisms with single-cell sequencing and spatial metabolomics, clarifying how SeNPs behave in bone niches. Additionally, the development of smart delivery systems—such as imaging-guided nanocarriers—and collaborative efforts between materials scientists, clinicians, and regulatory bodies will be essential to overcome scalability barriers and accelerate clinical adoption (112). Overcoming these barriers would position SeNPs as transformative agents in skeletal therapeutics, offering demonstrably safer and more efficacious treatment paradigms for affected patients.

## Glossary

A549: Adenocarcinomic human alveolar basal epithelial cells
ALP: Alkaline phosphatase
AMPKα/mTOR: 5’-AMP-activated protein kinase alpha/mammalian target of rapamycin
bFGF: Basic fibroblast growth factor
BMD: Bone mineral density
BMSC: Bone marrow mesenchymal stem cells
BMP-2: Bone morphogenetic protein 2
BRDs: Bone-related disorders
Caco-2: Human colorectal adenocarcinoma cell line
CC/Se-HAp: Carbon-coated selenium-hydroxyapatite
CDT: Chemodynamic therapy
COX-2: Cyclooxygenase-2
CZTS: Cadmium zinc telluride selenide
DI-SeNPs: Drimia indica-selenium nanoparticles
DOX: Doxorubicin
ERK: Extracellular signal-regulated kinase
FGFR: Fibroblast growth factor receptor
GPx1: Glutathione peroxidase 1
HA: Hyaluronic acid
hMSC: Human mesenchymal stem cell
HOS: Human osteosarcoma cells
ICG: Indocyanine green
IL-1β: Interleukin-1 beta
IL-6: Interleukin-6
JNK/FOXO3a: c-Jun N-terminal kinase/forkhead box O3a
KBD: Kashin-Beck disease
LNT-Se: Lentinan-selenium nanoparticles
LPS: Lipopolysaccharide
MC3T3-E1: Mouse pre-osteoblast cell line
MIC: Minimum inhibitory concentration
MMP13: Matrix metalloproteinase-13
MSQDs: Mesoporous quantum dots
NF-κB: Nuclear factor kappa-B
NFATc1: Nuclear factor of activated T-cells cytoplasmic 1
NK: Natural killer
NO: Nitric oxide
Nrf2: Nuclear factor erythroid 2-related factor 2
NSAIDs: Nonsteroidal anti-inflammatory drugs
OA: Osteoarthritis
OPG: Osteoprotegerin
OS: Osteosarcoma
PCL: Polycaprolactone
Pd@MSe-TPP: Palladium-manganese selenide-triphenylphosphonium nanomotors
Pd@Se-HA: Palladium-selenium-hyaluronic acid nanoparticles
PI3K/Akt: Phosphatidylinositol 3-kinase/Protein kinase B
PTT: Photothermal therapy
PVA: Polyvinyl alcohol
Qu-SeNPs: Quercetin-based selenium nanoformulation
RA: Rheumatoid arthritis
RANKL: Receptor activator of nuclear factor κB ligand
ROS: Reactive oxygen species
Runx2: Runt-related transcription factor 2
SD: Sprague-Dawley
Se: Selenium
SeCS: Selenium-chondroitin sulfate
SeCys₂: Selenocysteine
SeMetFa NPs: Selenium-Methionine-Folic Acid nanoparticles
SeMS: Selenium-doped mesoporous silica nanoparticles
SeNPs: Selenium-containing nanoparticles
SOD: Superoxide dismutase
SOD2: Superoxide dismutase 2
TGF-β: Transforming growth factor-beta
TNF-α: Tumor necrosis factor alpha
TrxR: Thioredoxin reductase
ZIF-8: Zeolitic imidazolate framework-8
ZnO: Zinc oxide.
